# Postmenopausal women's experiences of weight maintenance following a very low energy diet

**DOI:** 10.1002/osp4.654

**Published:** 2023-01-05

**Authors:** Claudia Harper, Judith Maher, Michelle Hsu, Anne Grunseit, Radhika Seimon, Amanda Sainsbury

**Affiliations:** ^1^ The Boden Collaboration for Obesity, Nutrition, Exercise, and Eating Disorders Faculty of Medicine and Health Charles Perkins Centre The University of Sydney Camperdown New South Wales Australia; ^2^ School of Health and Behavioural Sciences University of the Sunshine Coast Sippy Downs Queensland Australia; ^3^ Sydney School of Public Health Charles Perkins Centre Prevention Research Collaboration The University of Sydney Camperdown New South Wales Australia; ^4^ School of Human Sciences The University of Western Australia Crawley Western Australia Australia

**Keywords:** obesity, qualitative, very low energy total diet replacement, VLCD, VLED, weight loss, weight maintenance

## Abstract

**Introduction:**

Very low energy diets (VLEDs) effectively induce substantial weight loss in people with obesity, yet they are rarely used as a first line treatment. There is a belief that such diets do not teach the lifestyle behavior changes needed for long‐term weight maintenance. However, little is known about the lived experiences of people who have lost weight on a VLED in the long term.

**Methods:**

This study aimed to explore the behaviors and experiences of postmenopausal women who had followed a 4‐month VLED (using total meal replacement products [MRPs]), followed by a food‐based, moderately energy‐restricted diet for an additional 8 months, as part of the TEMPO Diet Trial. Qualitative in‐depth semi‐structured interviews were conducted with 15 participants at 12 or 24 months (i.e., at 8 or 20 months post diet completion). Transcribed interviews were analyzed thematically using an inductive approach.

**Results:**

Undertaking a VLED was reported by participants to confer advantages in weight maintenance that previous weight loss attempts had not been able to do for them. Firstly, the rapid and significant weight loss, in conjunction with ease of use, was motivational and helped instill confidence in the participants. Secondly, the cessation of a normal diet during the VLED was reported by participants to break weight gain‐inducing habits, allowing them to abandon unhelpful habits and to introduce in their place more appropriate attitudes toward weight maintenance. Lastly, the new identity, helpful habits and increased self‐efficacy around weight loss supported participants during weight maintenance. Additionally, participants reported that ongoing occasional use of MRPs provided a useful and easy new strategy for countering weight regain and supporting their weight maintenance regimen.

**Conclusion:**

Among the participants in this qualitative study, most of whom had maintained a loss of over 10% of their baseline body weight at the time of interview, using a VLED in the context of a clinical weight loss trial conferred confidence, motivation and skills for weight maintenance. These findings indicate that VLEDs with clinical support could be successfully leveraged to set up behaviors that will support weight maintenance in the long term.

## Graphical abstract

This study explored the weight maintenance behaviors and experiences of postmenopausal women who had followed a 4‐month VLED for weight loss. VLEDs in the context of this study—was reported by participants to confer advantages in weight maintenance that previous weight loss attempts had not been able to do for them. Using a VLED in the context of a clinical weight loss trial conferred confidence, motivation and skills for weight maintenance among participants. These findings show that VLEDs with clinical support could be successfully leveraged to set up behaviors that will support weight maintenance in the long term.

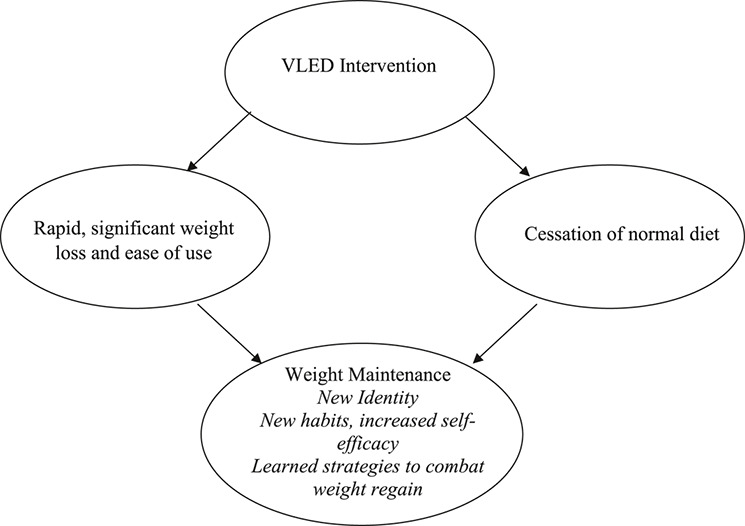

**Abbreviations**
VLCD: very low calorie diet
VLED: very low energy diet
VLETDR: very low energy total diet replacement

## 1 INTRODUCTION

To treat obesity, meta‐analyses indicate that the most effective long‐term non‐surgical, non‐pharmacological individual‐level weight loss treatments are very low energy diets (VLEDs).^1^, ^2^, ^3^ Internationally, VLEDs are defined by the Food Standards of Australia and New Zealand, the World Health Organization, the international CODEX standards of the WHO and Food and Agriculture Organization, as well as, the European Union as formula‐based foods used in weight control diets that provide between 2090 to 3350 kJ (500–800 kcal) daily.^4^, ^5^, ^6^, ^7^ This involves replacing all meals and snacks with pre‐packaged, nutritionally complete shakes or soups (to which water or milk is added), bars or other pre‐packaged meal replacement products (MRPs). Often, an allowance of low starch vegetables, black tea or coffee and no sugar products (e.g., diet drinks, diet jelly) is also used in this regimen.^8^, ^9^ The terms very low‐calorie diet (VLCD) and very low energy total diet replacement are sometimes used interchangeably with the term VLED. For clarity, we have used the term VLED throughout this paper, to comply with the most common international nomenclature.

VLEDs are safe in the short term and have been used for up to 5 months with no significant negative effects.^10^ They effectively induce fast weight loss in people with obesity.^11^ Furthermore, weight regain after these diets is no faster than after any other diet,^12^, ^13^, ^14^ and the greater initial weight loss after VLEDs compared to other diets has been shown to be predictive of long‐term maintenance of a lower body weight.^3^, ^12^, ^15^ Additionally, those who remain engaged with support after a VLED can retain clinically significant weight loss, of greater than or equal to 5% of initial body weight, in the long term (3 years).^16^ A meta‐analysis of long‐term weight maintenance, after either a VLED or a conventional food‐based diet, showed weight loss at 4–5 years after commencing the intervention of 6.6% of initial body weight versus 2.1%, respectively, demonstrating that greater long‐term weight loss can be achieved with a VLED.^3^ In a recent randomized controlled trial, participants who underwent a 16‐week VLED, compared to participants who underwent a conventional food‐based diet, were 2.6 times more likely (42% vs. 16%) to have lost 10% or more of their initial body weight at 3 years.^17^ Given that just 5% weight loss is known to confer metabolic advantages, VLEDs can also ameliorate obesity‐related chronic disease.

Despite the greater weight reductions and longer‐term maintenance of a reduced body weight, VLEDs are not routinely recommended as a first line weight loss method. Practice guidelines worldwide recommend gradual weight loss for overweight and obesity.^18^, ^19^, ^20^ The reasons for these recommendations are based on the assertion that gradual weight reduction in small increments is a more realistic goal, and making small changes slowly, confers better‐lasting weight maintenance results.^18^, ^19^, ^20^ Furthermore, healthcare professionals are reported to be reluctant to use VLEDs as a treatment for obesity.^21^, ^22^, ^23^, ^24^ While quantitative evidence shows better weight loss outcomes in the short‐ and long‐term, concerns from healthcare professionals about using VLEDs are often qualitative in nature. These concerns include beliefs that VLEDs may be difficult to adhere to, unpleasant to undertake, have low long‐term efficacy, and could promote weight cycling with concomitant potential negative psychological impact.^21^, ^22^, ^23^, ^24^ To address these qualitative concerns and inform healthcare professionals on how VLEDs are experienced by users, we undertook a systematic review of qualitative research on experiences during and after undertaking a VLED using total meal replacement.^25^, ^26^, ^27^, ^28^ Across all three studies available for inclusion in that review, VLED was generally reported by participants to be a positive experience, easy to follow, with decreased requirements to make decisions about food, and rapid weight loss increasing motivation for, and adherence to, the intervention.^28^ Another study on using a LED with total diet replacement for weight loss, also showed that total diet replacement is well tolerated by participants.^29^ Whilst these studies highlighted important aspects of using VLEDs for weight loss and reported that VLEDs and total diet replacement low energy diets are well tolerated in the specific populations studied, the significance of a VLED to the weight maintenance period was poorly described.^25^, ^26^, ^27^, ^28^

Therefore, this study was conducted to explore the experiences and behaviors of women who have undertaken a VLED intervention, with a specific focus on the weight maintenance phase. Acknowledgment by health researchers that the perspectives and experiences of participants and consumers have been neglected and are an important aspect of intervention acceptability, has progressed qualitative research as an important adjunct to quantitative findings of clinical research.^30^, ^31^, ^32^ Accordingly, this research was a sub‐study of the TEMPO Diet Trial (**T** ype of **E** nergy **M** anipulation for **P** romoting optimum metabolic health and body composition in **O** besity), a randomized controlled trial comparing the long‐term effects of severe energy restriction versus moderate energy restriction in postmenopausal women.^9^ The primary aim of the TEMPO Diet Trial was to make a head‐to‐head comparison of the long‐term (3‐year) effects of fast versus slow weight loss on body composition, including bone mineral density (an important predictor of osteoporotic fractures). Postmenopausal women were studied because the estimated lifetime risk of osteoporotic fractures is 2‐fold higher in women than in men (40% vs. 13%).^33^ The rationale and protocol of the TEMPO Diet Trial can be found in a previous publication.^9^

## 2 METHODS

### 2.1 Participants

This qualitative/exploratory study included 15 participants who were randomized to the severely energy‐restricted diet arm of the TEMPO Diet Trial. Participants had a mean (SD) age of 58.1 (3.8) years and mean (SD) body mass index of 34.0 (2.7) kg/m^2^ at baseline (i.e., at week 0, prior to commencement of the severely energy‐restricted diet), were at least 5 years postmenopausal at the time of recruitment, and sedentary (defined here as undertaking less than 3 h of structured physical activity per week). The full inclusion and exclusion criteria and the rationale for these have been detailed in our published protocol.^9^ The TEMPO Diet Trial and this qualitative sub‐study of the TEMPO Diet Trial were approved by the Sydney Local Health District Ethics Committee (Royal Prince Alfred Hospital Zone; [X12‐0081 & HREC/12/RPAH/135] and [X16‐0316 & HREC/16/RPAH/420], respectively) and the overall TEMPO Diet Trial was registered with the Australia and New Zealand Clinical Trials Registry (ACTRN12612000651886). All participants provided written informed consent prior to participation.

### 2.2 Severely energy‐restricted diet and weight maintenance diet

Details of the study protocol and 12‐month intervention for the severely energy‐restricted arm of the TEMPO Diet Trial have been reported in detail previously.^9^ Briefly, participants were randomized to four months (16 weeks) of severe energy restriction of 65%–75% relative to estimated energy expenditure, using a total meal replacement diet, followed by moderate energy restriction of 35%–25% for an additional 8 months (36 weeks, to a total of 12 months [52 weeks]), achieved with a food‐based diet based on the Australian Guide to Healthy Eating.^9^, ^34^

The severe energy restriction prescription for participants in this study consisted of three meal replacement shakes, two cups of non‐starchy vegetables, and one teaspoon of oil daily for 16 weeks. All MRPs (Kicstart^TM^ shakes and soups) were sourced from Prima Health Solutions Pty Ltd., Brookvale, NSW, Australia and were supplied to participants at no cost to them. The participants had regular one‐on‐one contact with a dietitian (approximately once every 2 weeks—both face to face and over the phone for the first 20 weeks), which decreased in intensity thereafter to approximately once monthly until 12 months, and then to once yearly after the 12‐month time point. Participants had the option of attending monthly face‐to‐face group support meetings after they had completed 12 months on the trial. Participants were then followed‐up at 24 and 36 months from the date they started the diet.

### 2.3 Recruitment

**FIGURE 1:**
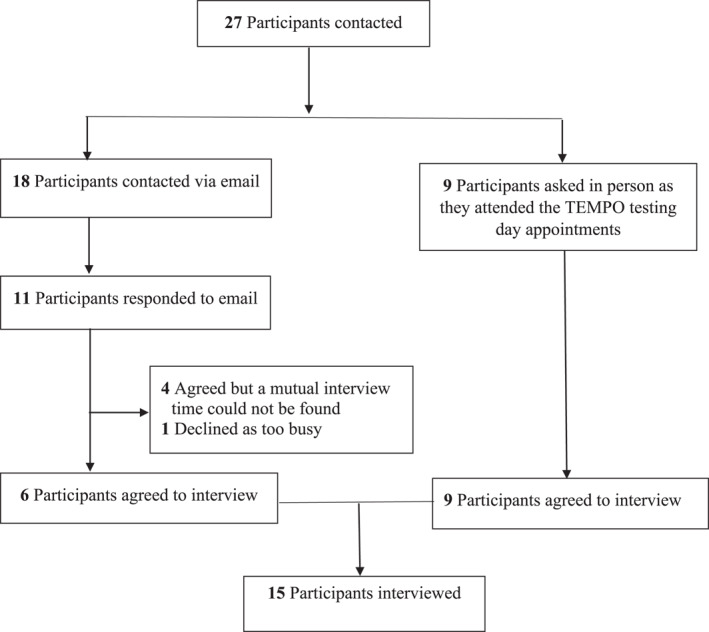
Recruitment of participants for this sub‐study of the TEMPO Diet Trial, and flow of participants through the sub‐study.

Women were eligible to participate in this sub‐study if they had completed the VLED intervention and had attended their appointment for follow up at either the 12‐ or 24‐month follow‐up timepoint for the clinical trial. A total of 27 participants meeting these criteria at the time of recruitment were contacted in two separate recruitment drives, either by email^17^ or in person^9^ when attending our clinical research facility for follow‐up data collection at their 12‐ or 24‐month time point (Figure 1). Of those, 15 participants were interviewed (Figure 1). Of the 18 participants contacted by email, 6 were interviewed, 4 agreed but a mutual time could not be found, 1 declined as too busy and 7 did not respond. Of the nine participants approached during their collection day, all nine agreed to be interviewed (Figure 1). Participants were interviewed either on or soon after the follow‐up time point at 12 months (11 participants) or 24 months (four participants). Recruitment of participants continued until saturation of data on the main concepts was achieved. This sample of 15 participants represents 35% of all 46 participants who completed the VLED arm of the TEMPO Diet Trial. Overall, only 4 out of 50 participants who were in the VLED arm of the trial discontinued prior to the 1‐year timepoint. Of the four who did not return at 1 year, two discontinued during the 16‐week VLED period.

### 2.4 Study design

This qualitative exploratory study was based on descriptive phenomenology,^35^, ^36^ as we explored both the common and uncommon experiences of postmenopausal women who had lost weight via a VLED and were attempting to maintain a reduced body weight. We also used research methods from grounded theory for interview questioning and to provide structure to the analytical process.^36^, ^37^

### 2.5 Data collection

Semi‐structured interviews were held at the Charles Perkins Centre Royal Prince Alfred Clinic on the University of Sydney campus in Camperdown, New South Wales, Australia, the same location where quantitative data were collected for the TEMPO Diet Trial, as this was convenient for both participants and researchers. Interviews were 1–1.5 h in duration, and were audio recorded. An interview discussion guide (Appendix A) was developed through discussions between researchers that included the TEMPO Diet Trial dietitians, chief investigators, and co‐authors with qualitative research experience, but who had no affiliation with the TEMPO Diet Trial. The interviews were conducted by a Masters student and a PhD student (and accredited practicing dietitian) who administered the weight loss intervention for 3 of the 15 participants (20%) in this sub‐study. All participants were encouraged to talk about their experiences, thoughts, beliefs and attitudes regarding their weight loss, undertaking a VLED intervention, and their weight maintenance efforts and experiences in the months after losing weight on a VLED. The interviews evolved over time. Concepts brought up by participants during earlier interviews were tested and expanded in later interviews. Data collection ceased when data saturation was reached (i.e., when no new major concepts around the topic were being discussed in the interviews).^38^

### 2.6 Data analysis

The interviews were transcribed verbatim and analyzed in‐depth using NVivo 11 qualitative data analysis software (QSR International Pty Ltd Version 11, 2016). Interviews were analyzed using line‐by‐line coding, rather than by imposing any theory or structure a priori, allowing the researchers to remain open to the possible emergence of themes not previously considered. Likenesses across codes were identified and checked for similarity in meaning before collapsing codes into categories. Categorization of codes allowed the researchers to attend to both commonly experienced aspects of the post weight loss journey, as well as outlying experiences. Apparently unique experiences were revisited within the context of the whole participants' narrative to better understand why it may have differed from other participants' experiences. Categories were then analyzed further to determine overarching themes that captured the essence of the lived experiences of these 15 participants in the VLED arm of the trial.

Once the broad themes had been developed, the researchers returned to the raw data within each theme to check that the actual experiences of the participants were well reflected in the overarching themes. The analyses were checked by two independent qualitative researchers, not affiliated with the TEMPO Diet Trial. Peer debriefing suggested that the amount of weight loss, and the amount of weight kept off at the time of the interview (i.e., weight maintenance) were relevant to the insight and experiences relayed by the participants. For this reason, the quantitative weight loss trajectories of the participants are shown in the results. Analysis of both outlying experiences as well as common experiences were reviewed in this context. The research findings are illustrated using quotes from participants. A COREQ Checklist is supplied in Appendix B.

## 3 RESULTS

### 3.1 Sample overview

Most of the 15 women participating in this sub‐study were in full‐time or part‐time employment, with only two of them being retired, albeit maintaining active volunteering roles. They all described a history of numerous previous weight loss attempts with gradual weight regain over their lifetimes. All participants could pinpoint at least one prior successful weight loss effort, either with a commercial weight loss program or via individual efforts. The most common commercial weight loss program previously used was Weight Watchers. The primary reasons stated for joining the current trial were health and weight loss. All 15 of the participants stated that joining a clinical trial in a university environment gave them a sense of safety and legitimacy about the dietary intervention, which some viewed as “drastic”. Participants also reported that being part of a clinical trial also increased their compliance and sense of responsibility to adhere to the intervention. None of the participants had attempted a total diet replacement before, however, all of them had either heard of meal replacements or had used them in an ad hoc manner prior to volunteering to be a participant in the TEMPO Diet Trial.

### 3.2 Weight loss results

**FIGURE 2:**
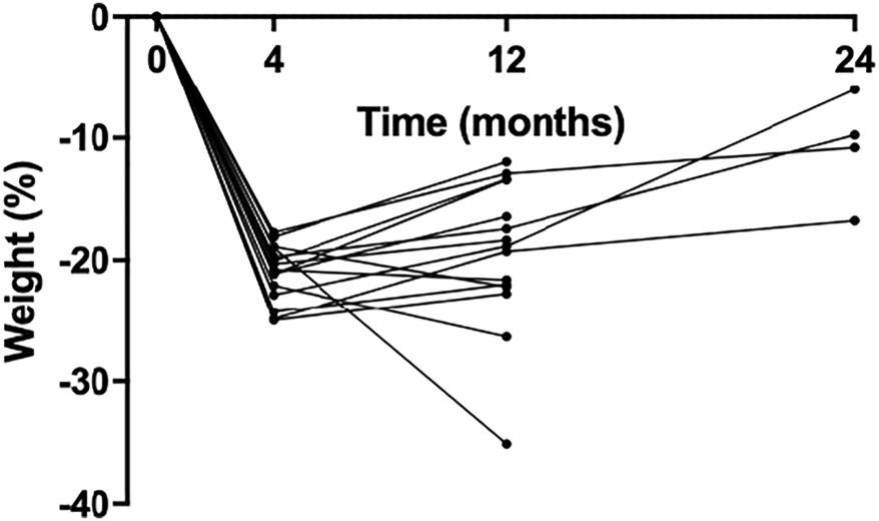
Weight change at 16 weeks and at time of interview (i.e., 12 or 24 months).

All participants exhibited significant weight loss while on the VLED, and all had maintained all or part of that weight loss at the time of their interview (i.e., at 12 or 24 months after VLED commencement), as shown in Figure 2. Moreover, at 12 months, 13 out of 15 (86.6%) participants in the current study, retained a weight loss of at least 10% of their baseline weight, as compared to 41 out of 46 (89.1%) for the whole group of participants who had been randomized to the severely energy‐restricted diet arm of the overall TEMPO Diet Trial. Additionally, the mean (SD) weight loss of this sample of participants closely matched the overall weight loss of the other 27 participants in the severely energy‐restricted diet arm (18.2 [2.5] kg vs. 17.2 [3.7] kg) (Table 1). Detailed weight loss results, as well as overall dropout rates at 12 months (4 out of 50 participants) for the TEMPO Diet Trial are available elsewhere.

**TABLE 1:** Age and Weight loss between groups

	Number	Age	Weight loss at 16 weeks	% Weight loss at 16 weeks
Participants who underwent interview	15	58.1 ± 3.8	18.2 ± 2.5	21.0 ± 2.4
Participants who were invited to be interviewed but who did not respond or declined interview	8	57.9 ± 3.5	15.5 ± 4.9	16.3 ± 4.2
All participants who were invited to be interviewed	27	58.3 ± 4.7	17.2 ± 3.9	22.0 ± 16.4

*Note*: Data are mean ± SD.

The mean (SD) weight loss at 16 weeks of the participants in this sub‐study compared with those who did not respond or who declined to participate (*n* = 8) was 18.2 (2.5) kg versus 15.5 (4.9) kg, respectively (Table 1). The weight gain at the time of interviews at 12 months in our participants versus those who did not respond or who declined to participate was 2.6 (6.5) kg versus 4.1 (5.4) kg, respectively. For ease of reading, individual weight losses are shown after the participant's identity code as a percentage of initial body weight at 4 months (end of the phase of the VLED intervention where total meal replacement diets were used), and at the time of the interview. For example, P01 (20.8% at 4 months, 21.6% at 12 months) shows that participant P01 had lost 20.8% of her initial (baseline or pre‐diet) body weight at the end of the 4‐month VLED, and 21.6% at 12 months, which was the time of her interview.

### 3.3 Overview of results—Weight maintenance

**FIGURE 3:**
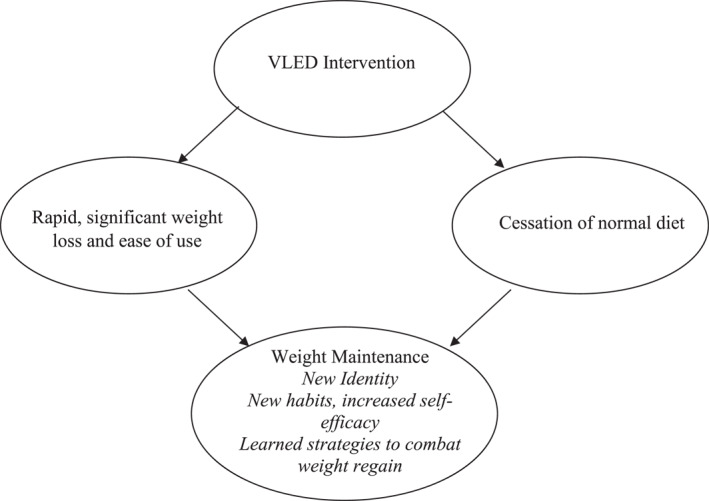
The three major themes and sub‐themes that emerged in our interviews of clinical trial participants about their experiences of the weight maintenance phase after a very low energy diet (VLED) achieved using meal replacement products.

This study revealed three major themes of how the VLED intervention—as experienced by the participants—influenced their subsequent approach to and thoughts about weight maintenance. These three themes are summarized in Figure 3. Two of these three major themes relate specifically to aspects of undertaking a VLED using meal replacements. These two themes are: Rapid, significant weight loss and ease of use of the VLED; and Cessation of a normal diet. These two aspects of the VLED were considered by participants to lead to the third of the three major themes, which we have termed “Weight maintenance”. This theme of Weight maintenance comprised three sub‐themes of experiences during the weight maintenance phase of the intervention. These three sub‐themes were: New identity; New habits and increased self‐efficacy; and Learned strategies to combat weight regain. In brief, the rapid, significant weight loss and ease of use of the VLED, in conjunction with the cessation of normal diet that was otherwise usually consumed by the participants, led to the perception of gaining a new identity, new habits and increased self‐efficacy, as well as having learned a strategy to combat weight regain during the weight maintenance phase of the intervention. These concepts are described below, in conjunction with participant quotes to give insight into the participants' viewpoints and feelings. Examples of further quotes from participants can be found in Appendix C.

#### 3.3.1 Rapid, significant weight loss and ease of use of the very low energy diet

Participants reported feeling buoyed by the rapid and continuous weight loss, which quickly induced noticeable changes in body size and/or shape, and this increased adherence throughout the 4 months of the VLED using MRPs. They also reported that the VLED protocol was easy to follow because they felt there was no planning, thinking or preparation required by comparison with other diets they had previously tried. These aspects of losing weight with a VLED using MRPs were described by the participants as being motivating and enjoyable.

P05 (18.9% at 4 months, 22.2% at 12 months) “*It would've taken me a year to lose the weight I lost in the four months, so to get quick results—was to get to a point where I felt like I was a normal weight.*”

#### 3.3.2 Cessation of normal diet

Participants reported feeling that the dramatic change in their eating patterns during the VLED was effective in breaking weight gain‐inducing habits and reconnecting with physiological feelings of fullness and true hunger. While experiencing the VLED with MRPs, and therefore the absence of any usual meals, snacks or drinks, the participants were able to recognize ingrained habits that had contributed to their inability to lose weight and keep it off previously. These previously unhelpful habits were brought into stark relief against the backdrop of cutting out all snacking and using shakes and soups as main meals. After the 4‐month VLED phase of the diet, participants reported taking steps to ensure what they had learned about themselves in that phase was put into practice.

P13 (22% at 4 months, 26.3% at 12 months) “*The VLED helped me recognize what I was doing that was wrong all the snacking the incidental eating, I wasn't eating proper meals […], I was just grabbing whatever, whenever and I had no idea what the feeling of fullness was like or the feeling of being hungry was like, so it has taught a lot. It has taught me all those bad habits; recognize those bad habits, it's allowed me to feel when I'm hungry and when I've had enough and when I can walk away. […] The thing that I really like now, that has helped sustain me is having that clean sweep. I got rid of bad habits that I didn't realize I had. It also allowed me, when I did start eating again, in that first week I had the most awful feeling of being too full. I really like the feeling of feeling like I can recognize when I'm hungry and just eat when I'm hungry.*”

### 3.4 Weight maintenance

#### 3.4.1 New identity

The large amount of weight lost in 4 months (mean [SD] of 18.2 [2.5] kg among the 15 participants in this study) instilled motivation to keep it off and inspired the participants to update their wardrobe in order to make their outer presentation more compatible with their inner perception of themselves. The participants spoke about finally being able to present themselves in a manner that they felt more comfortable with. This further increased the participants' desire to maintain their weight loss.

P06 (24.8% at 4 months, 16.8% at 24 months) “*I love clothes, I love fashion. I went out and bought a fortune's worth…you know, I look at fashion I look at people and how they look but I never really could participate in it and finally I could and it was so exciting, after all those years of not being able to […] and I sort of didn't even realize till after I lost the weight and the first time I walked in, saw a pair of pants hanging on a rack and thought ‘oh, that's so cute’. It was an outfit […] I tried them on, I almost exploded with joy.*”

#### 3.4.2 New habits and increased self‐efficacy

The participants reported that due to the experiences of losing weight with the VLED involving MRPs, they had new understandings and interpretations of their previous and current everyday practices. They continued to utilize what they had learned about weight loss, hunger, and the unhelpful beliefs they had prior to undertaking the VLED. This meant that new habits were formed, unhelpful habits that were highlighted during the cessation of their usual diet were not re‐introduced post VLED, and importantly, myths they had held around themselves, food and weight were banished.

P12 (24.9% at 4 months, 22.7% at 12 months) “*That's another thing I've learned, you know, I don't just think it's lunch time. I always say to myself now ‘Are you hungry?’ […] I say, ‘No, I'm not hungry’, well, that's like when you're on the diet, you weren't hungry, you don't need to eat.*”

The new habits and strategies increased the participants' confidence and self‐efficacy in being able to maintain weight and break unhelpful habits. Moreover, for those participants who had experienced previous unsuccessful weight loss attempts, the VLED addressed their beliefs that they were unable to lose weight and showed them that weight loss is a possibility if they tried. In those participants who had regained weight after the VLED, despite feeling disappointed, they reported retaining confidence in their abilities and the belief that weight loss is possible with direct actions. This new attitude to weight loss and regain, instead of reducing hope, acted to renew efforts to reduce weight regain and increased confidence that it would be successful.

P15 (22.9% at 4 months, 6.0% at 24 months) “*I'm disappointed, I'm angry, I'm frustrated all with myself because this is all totally controllable by me. […] I don't tend to be pessimistic my natural optimism is saying to me now, ‘you've done it before, you can do it again’.*”

#### 3.4.3 Learned strategies to combat weight regain

Along with new habits, all the participants reported continuing to use MRPs as part of their weight maintenance strategy. They felt it was a proven strategy that they can turn to for assistance in maintaining weight and optimism despite weight regain. The participants developed their own strategies to fit the use of MRPs into their individual lifestyles and preferences, and either used them intermittently to lose a couple of kilograms, or daily to maintain their reduced body weight.

P14 (17.7% at 4 months, 10.8% at 24 months) “*Knowing that I was going to the party yesterday I had two shakes during the day so that you know, I didn't load up on calories so I can enjoy the party.*”

P09 (19.0% at 4 months, 35.0% at 12 months) “*I just find the shakes a great tool. I can do things like if I want to do intermittent fasting to maintain things, the shakes will let me do that.*”

Overall, the participants reported that the VLED intervention had unique aspects which they had not experienced during other attempts at weight loss. These factors were highly rewarding and conferred a high level of motivation and self‐efficacy which persisted during weight maintenance. Recognizing bad habits during the VLED and changing unhelpful habits after the cessation of the VLED, combined with continued use of MRPs, helped the participants to feel empowered to maintain their weight loss for the long term.

## 4 DISCUSSION

Our research has extended previous findings and demonstrated that the rapid weight loss and the stark contrast of a VLED based on meal replacement compared with usual eating patterns provided motivation and new approaches to food which served to support participants in the weight maintenance phase. Further, the amount of weight lost by participants during the VLED allowed them to re‐engage with a positive perception of themselves, as signaled through new clothes and self‐image.

According to our interviewees, the rapid and significant weight loss they experienced conferred considerable motivation to not only adhere to the total meal replacement VLED, but also to continue to maintain that weight loss in the months after the VLED phase of the intervention had ended. This stands in contrast to worldwide Practice Guidelines for the management of obesity, which consistently recommend slow weight loss and small, achievable goals.^18^, ^19^, ^20^ For example, in Australia, the National Health and Medical Research Council (NHMRC) recommends that “a focus on sustainable (rather than restrictive) changes in dietary behavior may support motivation” and that a weight loss of 5% is a realistic and achievable goal.^19^ Setting “realistic” weight loss goals (less than 10% of initial body weight) prior to weight loss has been a cornerstone of weight loss interventions, however there is no evidence to support setting minimal weight loss goals and, setting the goal higher has been shown to be successful.^12^, ^14^ The commonly‐held assumption and oft cited recommendations^18^, ^19^, ^20^ that making small, slow changes over time is a better way to create more permanent lifestyle changes has not been supported by the narrative of our participants' experiences.

Buying a new wardrobe or being able to fit back into favorite items of clothing was described as a real and significant turning point by our participants, as well as an ongoing source of motivation for maintenance of reduced body weight. Shopping for clothing as a larger person can be confronting,^39^ and keeping favorite clothing has also been shown to be a motivator to commence weight loss efforts in women. In our study this was also a motivator for maintaining weight lost. The participants expressed positive self‐esteem and described how their new, slimmer self was far more aligned with the inner identity they held for themselves compared to their overweight body and larger clothing. Quality of life concepts such as self‐esteem have been shown to be lower among people living with obesity^40^ and weight loss, in particular significant weight loss, has been associated with positive effects of body image and self‐esteem.^41^ These quality‐of‐life factors seemed to coalesce in the participants in our study, who reported being finally able to express their true style and feel comfortable in their clothing.

Through choosing our daily wardrobe, we control the presentation of our identity, selfhood and well‐being. While identity shift has previously been identified as an enabler of weight loss maintenance,^42^ our research suggests participants' physical reality became aligned with their pre‐existing self‐identity through losing and maintaining weight lost. This served to reinforce their identity through being able to select clothing they liked and enjoyed wearing. Practical outcomes such as fitting into preferred clothing could be a goal‐setting point to either help people develop a desire to lose weight, or to continue to maintain a reduced body weight. We note that this perspective is very well represented, and seemingly well understood by commercial weight loss providers, as a legitimate goal or as a major theme of testimonials from their clients.

Despite our participants having tried to lose weight at least once—and in most cases, numerous times—previously, it seems the persistence of counterproductive habits remained, until meals and opportunities to eat were removed by the VLED and MRPs. This absence of their everyday food intake highlighted to participants a myriad of contexts in which they would reach for food, either consciously or unconsciously. These habits were newly understood as being unnecessary and detrimental to the participants' goal of permanent weight loss. For example, the assertion by our participants that they were for the first time recognizing what real hunger feels like, and that they are far more comfortable going without food than they had previously thought, made their previous failures to connect with their natural hunger cues more understandable. Taken together, participants' experiences and results during and after the VLED worked together to allow participants to critically evaluate their previous lifestyle habits and either cease old habits or introduce new habits, in order not to circumvent the results they had gained.

Identifying cues and triggers that lead to unhelpful eating habits is necessary in order to address unhelpful eating habits and develop alternative, intention‐driven habits.^43^ For example, knowing that stress or boredom leads a person to habitually reach for chocolate is considered to be a first step in eliminating that habit. Once identified, a disruption to that habit can be set in place to slowly cause the old habit to cease and a new habit to take its place. This is usually done by pre‐planning an alternative behavior in the same contextual situation that—with time and persistence—should replace existing undesirable habits.^44^ According to one estimate, it takes an average of 66 days to change an old habit or form a new habit.^45^ The VLED phase of the TEMPO Diet Trial being 4 months (120 days), presumably gave the participants time to recognize and eliminate unhelpful habits.

The VLED acted not only as an identifier and but also as a disruptor to unhelpful habits. Because the participants had to respond to usual triggers to snack or eat by using different (non‐eating) strategies for 4 months, this appeared to embed new responses to old triggers and forced the participants to actively re‐program behaviors that had previously contributed to their inability to lose weight. Additionally, recent analysis of food diaries of participants of the TEMPO Diet Trial has shown that diet quality improved for all participants from baseline, and this was mostly driven by a reduction in the intake of discretionary foods.^46^ The VLED intervention group also showed higher levels of physical activity and lower levels of sedentary behavior at 6 and 12 months, in comparison to the non‐VLED group.^47^ Taken together, these factors likely contribute to the longer‐term successful weight loss that is seen in response to VLEDs.

Participants continued to have feelings of self‐efficacy in their own ability to either maintain their reduced body weight (in those who were at a stable weight), or to re‐lose weight that had been regained (in those who had started to regain weight). Contrary to beliefs by healthcare professionals that VLEDs will induce yo‐yo dieting with negative psychological effects, we found that participants who had regained some of their weight continued to feel confident that they could successfully return to their goal weight, despite feeling disappointed about their weight regain. The success and discipline they had shown to themselves during the VLED conferred conviction that they had undertaken actions that could subsequently be repeated. While the majority of studies have shown an inverse relationship between higher number of past weight loss attempts and subsequent weight loss attempts, other studies have failed to show a relationship between these two variables.^48^, ^49^, ^50^

Although the number of previous weight loss attempts may have a deleterious effect on future efforts, a history of larger amounts of weight loss has shown to be associated with greater weight loss in subsequent attempts.^48^ This suggests that people who have lost large amounts of weight in the past retain higher levels of self‐efficacy and confidence for the success of future weight loss attempts.^48^ However, while it has been shown that higher self‐efficacy can be predictive of future successful weight loss attempts, and successful weight loss attempts can increase self‐efficacy, making weight maintenance more likely, to our knowledge, a continued feeling of confidence and self‐efficacy has not been researched or demonstrated in individuals who have subsequently re‐gained weight after a weight loss attempt.^51^, ^52^, ^53^, ^54^, ^55^ It is plausible however, as found by Myers et al.,^48^ that continuing high self‐efficacy among the participants in this research is due to the large amount of weight lost with a VLED, despite their weight re‐gain.

Finally, weight maintenance in our participants was supported by the ongoing use of MRPs, either as a daily supplement instead of a meal, or intermittently to lose weight or offset times of over‐indulging. The use of MRPs has shown to be helpful during weight maintenance,^56^ and is as effective as medication for maintaining a reduced body weight.^57^ Our participants chose to use MRPs of their own accord and purchased them from local retailers and found ways to use them effectively in the context of their own lifestyles. This appears to be a strategy that is easy to adopt, and that allows for easier maintenance of a reduced body weight.^58^, ^59^

To our knowledge, this is the first qualitative study to explore post‐VLED weight maintenance experiences. Given only three studies qualitatively explore the use of VLEDs to date, as outlined in a systematic review,^28^ this study added information not only about the use of VLED in a previously unstudied group (post‐menopausal women) but also, regarding the post‐diet weight maintenance phase. With consistent and known timing of interviews, the length of the weight maintenance phase is explicitly stated, and we were able to gather experiences of those who had maintained their weight and those who had regained weight after the VLED intervention.

Because our study was conducted in post‐menopausal Caucasian women, the results may not be transferable to other populations, including men. It is also possible, despite our sub‐study results closely resembling the whole group's weight loss results, that our respondents were participants who felt more positively about their experiences of weight maintenance. Indeed, the participants who were interviewed^3^ for this qualitative sub‐study had lost a mean of 2.7 and 5.6 kg more at 4 and 12 months respectively, compared to participants^8^ who either did not respond or declined to participate in this sub‐study. Further, our participants were part of a clinical trial which involved intensive support and strict inclusion criteria, and therefore their experiences may differ significantly from people using VLEDs for self‐directed weight loss and/or who would not have met the criteria for inclusion in the larger TEMPO Diet Trial.

## 5 CONCLUSION

Overall, the VLED intervention was well received and apparently easily executed by participants in this sub‐study. The significant weight loss increased motivation to maintain the reduced body weight post weight loss and demonstrated to participants that it was possible for them to lose weight. The radical departure from their usual dietary pattern helped to reveal to the participants counter‐productive habits, as well as ways to break those habits. Furthermore, increased self‐efficacy around weight loss strategies, and ongoing use of MRPs, supported future weight loss attempts and ongoing weight maintenance. This research will provide health care practitioners additional insights into the personal experiences of people undergoing a VLED intervention and provide nuance in situations, in ways not possible with quantitative data. Despite the negative beliefs held by community members and many healthcare professionals around fast weight loss and subsequent difficulty in maintenance of the reduced body weight, VLEDs can be an empowering and positive experience when used under clinical supervision in post‐menopausal women. Future research could examine if these experiences translate to broader populations.

## AUTHOR CONTRIBUTIONS

For this paper Claudia Harper made the following contributions: conceived the idea for the paper; obtained ethics approval; conducted interviews; conducted all the analysis; interpreted the findings; and wrote all sections of the manuscript. Michelle Hsu contributed to partial interviewing of participants; Anne Grunseit and Judith Maher provided input to analysis and interpretation of findings; Amanda Sainsbury supervised the writing of manuscript; all co‐authors provided feedback during editing and final manuscript.

## CONFLICT OF INTEREST

RVS reported serving on the Nestlé Health Science Optifast VLCD advisory board. AS reported owning 50% of the shares in Zuman International, which receives royalties for books she has written and payments for presentations at industry conferences; receiving presentation fees and travel reimbursements from Eli Lilly and Co, the Pharmacy Guild of Australia, Novo Nordisk, the Dietitians Association of Australia, Shoalhaven Family Medical Centres, the Pharmaceutical Society of Australia, and Metagenics; and serving on the Nestlé Health Science Optifast VLCD advisory board from 2016 to 2018. MH reported working for Prima Health Solutions in 2018–2019. No other disclosures were reported.

## APPENDIX A

1. What brought you to this trial? What made you want to go on the trial?
2. Where did you see it advertised? Have you heard of these before? What was your impression of the VLED?
3. What made you decide to participate? Did you have any health concerns which prompted you to want to lose weight?
4. Can you tell me about previous dieting/weight regain experiences? What do you think the factors are that contributed to you being overweight?
5. Expectations: what did you expect to get out of the trial when you started?
6. What kind of weight loss did you expect? And did your weight loss match your expectations?
7. What was it like when you got randomized to the VLED?
8. Please walk me through a typical day on the VLED or AGHE (you mentioned XXX, why did you do that?) Specific examples:
  – (living with other people) how do you manage the cooking/shopping etc.
  – How about weekends, what's that like?
  – Social events: how did you manage it? and then what happens?
9. What are some of the upsides of the VLED? What were the downsides?
10. What did you find hard or easy about being on the VLED diet? If you could change anything about trying to lose weight on a VLED, what would it be?
11. How hard is your diet now and how hard was it before the trial?
  – What about the VLED/trial made you feel/think XX?
12. What did you learn about yourself from going through the VLED? Did the VLED change your eating habits or help you break any habits? Has that continued for you after coming off the diet?
13. Have you changed anything because of this experience?
14. Wrap up
15. What have you gotten out of the trial?/what has the trial taught you?/What have you learned from the this? Overall, how was your experience?
  – Are you glad you joined? What makes you say that?
  – What could you have improved on? How would you have done that?
  – What would have made things easier? Why do you think these factors would help?
16. Weight maintenance
17. What aspects of weight maintenance have you found most difficult?
18. Is there anything you learned while being on the trial diet that has helped you with weight maintenance?
19. Do you have any tips or trick to give about weight maintenance?
20. Is there any else that you would like to tell me about your experience?
21. Is there anything that I may have missed which you think is important and you would like to tell me?

## APPENDIX B

**Manuscript**: Postmenopausal women's experiences of weight maintenance following a VLED.

**Consolidated criteria for reporting qualitative studies (COREQ): 32‐item checklist**

Developed from:

Tong A, Sainsbury P, Craig J. Consolidated criteria for reporting qualitative research (COREQ): a 32‐item checklist for interviews and focus groups. Int J Qual Health Care. 2007;19(6):349–357.

**Table:**

No. Item	Guide questions/description	Reported on page #
Domain 1: Research team and reflexivity		
Personal characteristics		
1. Interviewer/facilitator	Which author/s conducted the interview or focus group?	Page 9—Methods CH&MH
2. Credentials	What were the researcher's credentials? For example, PhD, MD	Page 9—CH‐APD + PhD candidate
		MH‐Masters student
3. Occupation	What was their occupation at the time of the study?	Page 9—CH‐APD + PhD candidate
		MH‐Masters student
4. Gender	Was the researcher male or female?	Page 1—Female
5. Experience and training	What experience or training did the researcher have?	Page 9—Methods CH‐Previous Qual Honors project.
		TEMPO Team
		JM‐Qual researcher
		AG‐Qual researcher
		Page 10—Independent Qual researchers (JM &AG)
Relationship with participants		
6. Relationship established	Was a relationship established prior to study commencement?	Page 8—Methods Relationship established by being part of TEMPO Trial—Numerous contacts with TEMPO team throughout TEMPO RCT
7. Participant knowledge of the interviewer	What did the participants know about the researcher? For example, personal goals, reasons for doing the research	Page7 & 8—All participants were given an Information sheet to read and signed a consent form.
8. Interviewer characteristics	What characteristics were reported about the inter viewer/facilitator? For example, Bias, assumptions, reasons and interests in the research topic	Page 6—Reasons study was done as a qual. Sub study as part of larger TEMPO Trial. Also Page 8
Domain 2: Study design		
Theoretical framework		
9. Methodological orientation and Theory	What methodological orientation was stated to underpin the study? For example, grounded theory, discourse analysis, ethnography, phenomenology, content analysis	Page 9—Methods
Participant selection		
10. Sampling	How were participants selected? For example, purposive, convenience, consecutive, snowball	Page 8 & 9—Methods
11. Method of approach	How were participants approached? For example, face‐to‐face, telephone, mail, email	Page 8 & 9—Methods
12. Sample size	How many participants were in the study?	Page 8—Methods
13. Non‐participation	How many people refused to participate or dropped out? Reasons?	Page 8 & 31—(Figure 1)
Setting		
14. Setting of data collection	Where was the data collected? For example, home, clinic, workplace	Page 9—Methods
15. Presence of non‐participants	Was anyone else present besides the participants and researchers?	Page 9—Inferred as one to one interviews
16. Description of sample	What are the important characteristics of the sample? For example, demographic data, date	Page 7 and ref. to TEMPO Study rationale and protocol published paper
Data collection		
17. Interview guide	Were questions, prompts, guides provided by the authors? Was it pilot tested?	Page 9 and Appendix A and page 33
18. Repeat interviews	Were repeat interviews carried out? If yes, how many?	No, inferred on page 9
19. Audio/visual recording	Did the research use audio or visual recording to collect the data?	Page 9—Methods
20. Field notes	Were field notes made during and/or after the interview or focus group?	Page 9—Methods
21. Duration	What was the duration of the inter views or focus group?	Page 9—Methods
22. Data saturation	Was data saturation discussed?	Page 10—Methods
23. Transcripts returned	Were transcripts returned to participants for comment and/or correction?	No
Domain 3: Analysis and findings		
Data analysis		
24. Number of data coders	How many data coders coded the data?	Page 10—Data analysis
25. Description of the coding tree	Did authors provide a description of the coding tree?	Page 10
26. Derivation of themes	Were themes identified in advance or derived from the data?	Page 10
27. Software	What software, if applicable, was used to manage the data?	Page 10
28. Participant checking	Did participants provide feedback on the findings?	No
Reporting		
29. Quotations presented	Were participant quotations presented to illustrate the themes/findings? Was each quotation identified? For example, participant number	Page 13 to 20—Results & Appendix C
30. Data and findings consistent	Was there consistency between the data presented and the findings?	Page 13–20—Results & Appendix C
31. Clarity of major themes	Were major themes clearly presented in the findings?	Yes. They were.
		From page 13–20 and Figure 3 (p. 33)
32. Clarity of minor themes	Is there a description of diverse cases or discussion of minor themes?	Discussion of major and minor themes
		From page 13–20

## APPENDIX C

**Table:**

Major themes	Quotes
Rapid, significant weight loss and ease of use of the VLED.	**P04** (23.4% at 4 months, 21.6% at 12 months) “I felt good, it was the easiest diet I've ever been on—you know, I've done Weight Watchers, I've done those sorts of things over the years, but that… you know, the fact that I didn't have to think about food, except for my two cups of vegetables a day and that was pretty easy, it was very easy, yep, I really enjoyed being on the diet.”
	**P02** (20.3% at 4 months, 18.4% at 12 months) “I didn't have a reason to want to break it, because also when you start looking at the scales go down, that's the motivation too. It's quite remarkable.”
	**P09** (19.0% at 4 months, 35.0% at 12 months) “Yes, hugely motivating. That's one of the things that's so fantastic about the shakes and soups is that it is quick—you get a result and so you think, ‘oh, I can do this!’, you get the rewards straight away.”
	**P05** (18.9% at 4 months, 22.2% at 12 months) It would've taken me a year to lose the weight I lost in the 4 months, so to get quick results—was to get to a point where I felt like I was a normal weight.”
Cessation of normal diet	**P10** (18.0% at 4 months, 12.0% at 12 months) “I kept myself sort of propped up all the time before but yeah, so to actually feel that feeling, that you're hungry, yeah it's only a more recent occurrence.” […] “I would sometimes eat before I got hungry. […] I was getting into that kind of mindset where I was really eating before I should, so I recognized that and when I was on the shakes, I realized ‘Oh, for heaven's sake, you're not going to die, you know of lack of food’, and it was really good just to know how little you can survive on […]. That was quite liberating because I felt that I had got myself in a little rut with food.”
	**P07** (20.0% at 4 months, 13.4% at 12 months) “The longer that I stayed on the VLED, the easier it got […]. Not pushing me to go and look for food—the habits again, ‘ok, it's mid‐afternoon, I might be hungry,’ but the longer I stayed on it, the less I wanted to do that. So again, it's probably just breaking the habit.” […] “It's a habit, it's, ‘I'll just get up and have a cup of coffee or I'll have a quick snack’ and you go ‘No! I can't do that’ (laughs). […] ‘What am I going to do?’ I get up and walk up and down the verandah.” D1 “For some people it just kickstarts that weight loss, which then gives them the motivation to keep going.”
	**P13** (22% at 4 months, 26.3% at 12 months) “The VLED helped me recognize what I was doing that was wrong all the snacking the incidental eating, I wasn't eating proper meals […], I was just grabbing whatever, whenever and I had no idea what the feeling of fullness was like or the feeling of being hungry was like, so it has taught a lot. It has taught me all those bad habits; recognize those bad habits, it's allowed me to feel when I'm hungry and when I've had enough and when I can walk away. […] The thing that I really like now, that has helped sustain me is having that clean sweep. I got rid of bad habits that I didn't realize I had. It also allowed me, when I did start eating again, in that first week I had the most awful feeling of being too full. I really like the feeling of feeling like I can recognize when I'm hungry and just eat when I'm hungry.”
Weight maintenance	***New identity***
	**P02** (20.3% at 4 months, 18.4% at 12 months) “I've lost the great big chunk of weight, gosh, I would have no desire to put that weight back on, so that's really—oh heavens, I just would not do that. […] You know, my clothes are fitting well, all of those things mean something to me.”
	**P11** (21.0% at 4 months, 13.4% at 12 months) “All the old clothes—all the bigger clothes that I eventually threw out are gone and the stuff that I bought is still fitting me—I'm still wearing it. So, it's really nice when your clothes are comfortable, that makes you feel better I think.”
	**P06** (24.8% at 4 months, 16.8% at 24 months) “I love clothes, I love fashion. I went out and bought a fortune's worth…you know, I look at fashion I look at people and how they look but I never really could participate in it and finally I could and it was so exciting, after all those years of not being able to […] and I sort of didn't even realize till after I lost the weight and the first time I walked in, saw a pair of pants hanging on a rack and thought ‘oh, that's so cute’. It was an outfit […] I tried them on, I almost exploded with joy.”
	***New habits and increased self‐efficacy***
	**P04** (23.4% at 4 months, 22.0% at 12 months) “I think, again, to do with habits, before I went on the diet, I tended to eat snacks and stuff between meals, that's one of the habits I've broken so I don't have morning tea now, I don't have afternoon tea. I've changed a lot of bad habits.”
	**P11** (21.0% at 4 months, 13.4% at 12 months) “I can avoid eating at all, at morning tea, if it's someone's birthday, so I'm better at that and that becomes a habit for me, if I'm in the habit of doing it I don't even think about it.”
	**P09** (19.0% at 4 months, 35.0% at 12 months) “Instead of taking the train to […], I'll take the train to […] and walk home and that will give me some more steps. That has changed now too, I'll tell you—to see missing the train as a positive to get some extra exercise instead of thinking ‘What a drag, I'll have to wait’.”
	**P03** (21.1% at 4 months, 16.2% at 12 months) “I think you have to accept that you really have to modify the way you live your life, because, you know, I developed a pattern of eating that wasn't helping me, I was eating on the go all the time and I was eating when I was tired, and I've got to make a decision that if I choose to be slim then I have to change the way I do things.”
	**P12** (24.9% at 4 months, 22.7% at 12 months) “That's another thing I've learned, you know, I don't just think it's lunch time. I always say to myself now ‘Are you hungry?’ […] I say, ‘No, I'm not hungry’, well, that's like when you're on the diet, you weren't hungry, you don't need to eat.”
	**P02** (20.3% at 4 months, 18.4% at 12 months) “I feel more confident in knowing that I have got these strategies, because […] before the diet, I wouldn't have identified these strategies to be able to break the habit—so that's what I've learned.”
	**P08** (19.8% at 4 months, 9.7% at 24 months) “I mean it's definitely the major benefit is it (the VLED) proved to me that I can make it (weight loss) happen. It's proved to me if I put the effort in, I can make it happen—I'm now even more motivated and I know I can do it […]. and so it just shows me I have to try harder.”
	**P11** (21.0% at 4 months, 13.4% at 12 months) “It makes you realize that I can do it again—that I can do it—it's not impossible.”
	**P09** (19.0% at 4 months, 35.0% at 12 months) “Then when I saw it (the weight) coming off I thought, ‘Yes, I can do this!’ Having lost the weight, for me, reinforces that if I set a goal, I will do it. And I feel good about that. And also, that I can influence my health.”
	**P15** (22.9% at 4 months, 6.0% at 24 months) “I'm disappointed, I'm angry, I'm frustrated all with myself because this is all totally controllable by me. […] I don't tend to be pessimistic my natural optimism is saying to me now, ‘you've done it before, you can do it again’.”
	**P07** (20.0% at 4 months, 13.4% at 12 months) “[The VLED taught me] the discipline, that if I make a decision to do something, I can do it.”
	**P03** (21.1% at 4 months, 16.2% at 12 months) “I feel confident again, I can do it easily.”
	***Learned strategies to combat weight regain***
	**P06** (24.8% at 4 months, 16.8% at 24 months) “I have gone on the shakes like for a week at a time, just the shakes, just to kind of maybe knock off a couple kilos quickly and efficiently, and it's a great feeling.”
	**P01** (20.8% at 4 months, 21.6% at 12 months) “And I still have a shake for breakfast now, I've kept that up.”
	**P14** (17.7% at 4 months, 10.8% at 24 months) “Knowing that I was going to the party yesterday I had two shakes during the day so that you know, I didn't load up on calories so I can enjoy the party.”
	**P09** (19.0% at 4 months, 35.0% at 12 months) “I just find the shakes a great tool. I can do things like if I want to do intermittent fasting to maintain things, the shakes will let me do that.”
